# Analysis of thyroid involvement in children and adult Langerhans cell histiocytosis: An underestimated endocrine manifestation

**DOI:** 10.3389/fendo.2022.1013616

**Published:** 2022-09-30

**Authors:** Yuanmeng Li, Long Chang, Xiaofeng Chai, He Liu, Hongbo Yang, Yu Xia, Li Huo, Hui Zhang, Naishi Li, Xiaolan Lian

**Affiliations:** ^1^ Department of Endocrinology, Key Laboratory of Endocrinology of National Health Commission, Peking Union Medical College Hospital, Chinese Academy of Medical Sciences and Peking Union Medical College, Beijing, China; ^2^ State Key Laboratory of Complex Severe and Rare Diseases, Peking Union Medical College Hospital, Chinese Academy of Medical Sciences and Peking Union Medical College, Beijing, China; ^3^ Department of Hematology, Peking Union Medical College Hospital, Chinese Academy of Medical Sciences and Peking Union Medical College, Beijing, China; ^4^ Department of Ultrasound, Peking Union Medical College Hospital, Chinese Academy of Medical Sciences and Peking Union Medical College, Beijing, China; ^5^ Department of Nuclear Medicine, Peking Union Medical College Hospital, Chinese Academy of Medical Sciences and Peking Union Medical College, Beijing, China; ^6^ Department of Clinical Pathology, Peking Union Medical College Hospital, Chinese Academy of Medical Sciences and Peking Union Medical College, Beijing, China

**Keywords:** Langerhans cell histiocytosis, thyroid involvement, diabetes insipidus, fine needle aspiration biopsy, thyroid ultrasonography, pathological diagnosis, 18-F-fluorodeoxyglucose positron emission tomography/computed tomography

## Abstract

**Background:**

Langerhans cell histiocytosis (LCH) is a rare disease caused by the clonal expansion of CD1a+/CD207+ LCH cells. The thyroid involvement in LCH has mostly been described in case reports.

**Methods:**

We retrospectively evaluated the clinical characteristics, diagnosis, and treatment of 27 children and adult patients with thyroid LCH in our center between 2010 and 2021.

**Results:**

The incidence of thyroid LCH was 14.00% (7/50) in children and 10.10% (20/198) in adults, respectively. Among patients with thyroid involvement, 81.5% presented with diabetes insipidus (DI) as the first symptom, and 51.9% complained of neck swelling or mass. Children and adults with thyroid LCH had higher frequencies of the hypothalamic-pituitary axis (HPA) (children: 100% vs. 62.8%, P=0.05; adult: 95% vs. 42.1%, P<0.001), the lung (children: 85.7% vs. 25.6%, P=0.004; adult: 70% vs. 50.6%, P=0.099), and a lower frequency of bone (children: 14.3% vs. 55.8%, P=0.049; adult: 45% vs. 73.6%, P=0.008) involvement than patients without thyroid involvement. Patients with thyroid LCH had a higher frequency of primary hypothyroidism and a lower frequency of euthyroidism than patients without it. The two major types of ultrasound imaging were diffuse (55%) and nodular type (45%). The standardized uptake value of thyroid on 18-F-fluorodeoxyglucose positron emission tomography/computed tomography was 5.3–12.8. The diagnoses were confirmed using thyroid aspiration (54.5%) or surgery (45.5%). In addition, thyroid LCH combined with papillary thyroid carcinoma was not rare (2/27).

**Conclusion:**

Thyroid involvement in LCH is not rare. Furthermore, identifying thyroid involvement can facilitate the pathological diagnosis of LCH. Therefore, the possibility of thyroid LCH should be fully investigated in patients with DI, primary hypothyroidism, abnormal thyroid ultrasound results, and multi-system disease. In addition, thyroid aspiration can confirm suspected thyroid LCH. Finally, special attention should be paid to evaluating HPA and pulmonary involvement in thyroid LCH.

## Introduction

Langerhans cell histiocytosis (LCH) is a disease driven by misguided myeloid differentiation, with the feature of accumulated CD1a+/Langerin+ Langerhans cells in lesions (1, 2). It is more common in children than adults, and its annual incidence is 2.6–8.9 cases per million in children younger than 15 years (2, 3). LCH can be classified into single-system and multi-system diseases based on lesions site, and the number of involved sites, among which the skeletal involvement is the most common, and the liver, spleen, and hematopoietic system are at-risk (of mortality) organs (4). In addition, involvement of the lung, skin, and endocrine system are also frequently observed in LCH.

LCH treatment is risk-adapted; local therapy or simple observation is suitable for single-site diseases, whereas multi-system diseases require systemic treatment. However, a considerable number of patients experience recurrence after treatment.

As a component of LCH, endocrine involvement has a particular predilection for the hypothalamic-pituitary axis (HPA). HPA abnormalities in LCH have been extensively described in several studies (5, 6). In contrast, mainly a few case reports focused on thyroid involvement. Therefore, this study aimed to report the prevalence of thyroid involvement in LCH, summarize its clinical manifestations, laboratory findings, ultrasonographic features, and diagnostic process, and propose the importance of identifying thyroid involvement in optimizing the pathological diagnosis of LCH.

## Materials and methods

The medical records system of Peking Union Medical College Hospital was accessed to retrieve information of patients hospitalized and diagnosed with LCH between March 2010 and March 2021, and those with thyroid involvement were selected. All 27 patients presented clear pathological evidence for LCH diagnosis. The following data were extracted from the medical records: general information, disease manifestations, laboratory findings, thyroid ultrasound findings, 18-F-fluorodeoxyglucose positron emission tomography/computed tomography (18-F-FDG PET/CT) findings, pathology, diagnostic modality, treatment, and prognosis. The study was approved by the Peking Union Medical College Hospital Ethics Committee (No. I-22PJ108). The study was performed in accordance with the No. I Declaration of Helsinki as revised in 2013.

Primary hypothyroidism was defined as an elevated thyroid-stimulating hormone (TSH) level and a decreased free thyroxine (FT4) level, subclinical hypothyroidism was defined as an elevated TSH level and a normal FT4 level, and secondary hypothyroidism was defined as decreased TSH and FT4 levels. In addition, fine needle aspiration biopsy was defined as a biopsy performed with a needle with a gauge of 23G, while core needle biopsy was defined as a biopsy performed with a needle with a gauge of 18G.

### Statistical analyses

All analyses were performed using SPSS statistics version 23.0 (SPSS Inc., Chicago, IL, USA). The Mann–Whitney U-test compared continuous variables between groups. Categorical variables were reported as percentages and were analyzed using Fisher’s exact test. A p-value of <0.05 was considered significant.

## Results

### Patients

There were 248 patients with LCH during the study period, including 50 children (younger than 18 years) and 198 adults. Twenty-seven patients with thyroid involvement were identified, accounting for 10.89% of all LCH patients. The incidence of thyroid LCH was 14.00% (7/50) and 10.10% (20/198) in children and adults, respectively. Thyroid involvement was pathologically diagnosed in 22 cases, and diagnosis in the remaining 5 was mainly based on elevated FDG accumulation of thyroid lesions on 18-F-FDG PET/CT. The detailed characteristics of all 27 patients with thyroid LCH are summarized in **Table 1**. In addition, we compared the characteristics of LCH patients with and without thyroid lesions separately for children and adults (**Table 2**).

**Table 1:** The characteristics, treatment, and prognosis of patients with thyroid LCH.

Case number	Patient age (years old) /gender	Other organinvolvement	Laboratory findings	Ultrasound imaging type	18-F-FDG PET/CT (SUVmax)	Treatment	Survival
Case 1	8/Male	HPA, lung, LN	Subclinical hypothyroidism: FT4 1.40 ng/dL, TSH 14.5 μIU/mL; TPO-Ab (-), TG-Ab (-); Tg 516.7 ng/mL	Diffuse type	N	Vindesine, prednisone, methotrexate	No evidence of recurrence after 5 years
Case 2	13.5/Male	HPA	Euthyroidism: FT4 1.48 ng/dL, TSH 2.7 μIU/mL; TPO-Ab (-), TG-Ab (-)	Nodular type	N	Lost follow-up	Lost follow-up
Case 3	14.4/Male	HPA, lung	N	Diffuse type	8.3	MA*6	No signs of active disease after 2 years
Case 4	15/Male	HPA, lung, LN	Primary hypothyroidism: FT4 0.51 ng/dL, TSH 14.114 μIU/mL; TPO-Ab (+), TG-Ab (+);	Nodular type	N	CVOP*5	No signs of active disease after3 years
Case 5	15.2/Female	HPA, lung, skin	Primary hypothyroidism: FT4 0.70 ng/dL, TSH 8.21 μIU/mL; TPO-Ab (-), TG-Ab (+); Tg 1.96 ng/mL	Diffuse type	Elevated (unknown value)	MA*6	No signs of active disease after 5 years
Case 6	16/Female	HPA, lung, LN, gastrointestinal tract, pancreas, liver	Primary hypothyroidism: FT4 0.64 ng/dL, TSH 15.4 μIU/mL	N	Elevated (unknown value)	CEOP*3, other chemotherapy drugs(unknown)	Recurrence after 2 years
Case 7	16.7/Male	HPA, lung, LN, bone, skin, pericardial effusion	TPO-Ab (-), TG-Ab (-)	N	N	Radiotherapy (neck lesion), MA*6	No signs of active disease after 2 years 6 months
Case 8	19/Female	HPA, LN, breast	Secondary hypothyroidism: FT4 0.71 ng/dL, TSH 0.226 μIU/mL; normal Tg (unknown value)	N	Elevated (unknown value)	Partial thyroidectomy, followed by low-dose cytarabine	No signs of active disease after 1 year 5 months
Case 9	20/Female	HPA, lung, LN, bone, skin, thymus	Primary hypothyroidism: FT4 0.69 ng/dL, TSH 12.631 μIU/mL; TPO-Ab (-), TG-Ab (-)	Nodular type	N	Subtotal thyroidectomy, followed by CEOP*8 & GDP-ML*1	Recurrence after 7 months, treated with cytarabine, cladribine, lenalidomide and TCD, alive after 11 years
Case 10	20/Female	lung, LN, bone, liver, spleen	Primary hypothyroidism: FT4 0.77 ng/dL, TSH 16.046 μIU/mL; TPO-Ab (+), TG-Ab (+)	N	Elevated (unknown value)	Vinblastine, cytarabine, dexamethasone	Progressed → CEOP → cladribine & cytarabine →TCD, alive for 9 years
Case 11	21/Male	HPA, lung	Primary hypothyroidism: FT4 0.423 ng/dL, TSH 11.54 μIU/mL;	Nodular type	N	MA*6	Alive after 8 years
Case 12	22/Male	HPA, lung, LN, bone	Subclinical hypothyroidism: FT4 1.346 ng/dL, TSH 4.949 μIU/mL; TPO-Ab (-), TG-Ab (-); Tg 113.8 ng/mL	Diffuse type	10.8	MA*6	Lost follow-up
Case 13	22/Male	HPA, lung, LN, bone, otitis externa	Primary hypothyroidism: FT4 0.62 ng/dL, TSH 18.58 μIU/mL; TPO-Ab (-), TG-Ab (-); Tg 3.7 ng/mL	Diffuse type	N	Total thyroidectomy, followed by MA*6	No evidence of recurrence after 9 years
Case 14	22/Male	HPA, lung, liver	Subclinical hypothyroidism: FT4 1.085 ng/dL, TSH 9.146 μIU/mL; TPO-Ab (-), TG-Ab (-); Tg 644.6 ng/mL	Diffuse type	Elevated (unknown value)	Lost follow-up	Lost follow-up
Case 15	22/Female	HPA, LN, bone, gum, spleen	N	N	N	Subtotal thyroidectomy, followed by medium-dose cytarabine	Lost follow-up
Case 16	23/Male	HPA, lung, liver	Secondary hypothyroidism: FT4 0.77 ng/dL, TSH 0.355 μIU/mL; TPO-Ab (-), TG-Ab (-)	Diffuse type	8.5	MA*6	Recurrence after 1 years, treated with TCD and dabrafenib, alive after 4 years 10 months
Case 17	24/Male	HPA, LN, bone, skin, thymus, liver	Euthyroidism: FT4 1.2 ng/dL, TSH 2.539 μIU/mL	N	5.3	Radiotherapy (bone lesion), 6×CEOP	Progressed after CEOP → medium-dose cytarabine →TCD, alive for 3 years 6 months
Case 18	24/Female	HPA, lung	Euthyroidism: FT4 1.534 ng/dL, TSH 3.968 μIU/mL; TPO-Ab (-), TG-Ab (-)	Diffuse type	12.8	MA*6	No signs of active disease after 5 years
Case 19	29/Male	HPA, lung, liver	Euthyroidism: FT4 1.255 ng/dL, TSH 2.679 μIU/mL; TPO-Ab (-), TG-Ab (-)	Nodular type	Elevated (unknown value)	MA*3, ongoing TCD	No signs of active disease after 3 years
Case 20	35/Female	HPA, lung, otitis externa	Euthyroidism: FT4 0.856 ng/dL, TSH 0.947 μIU/mL; TPO-Ab (-), TG-Ab (+); Tg 104 ng/mL	Diffuse type	9.26	MA*6	Recurrence after 1 years, treated with TCD, alive after 4 years
Case 21	36/Male	HPA, lung	Euthyroidism: FT4 1.353 ng/dL, TSH 0.561 μIU/mL; TPO-Ab (-), TG-Ab (-)	Diffuse type	N	MA*6	No evidence of recurrence after 4 years
Case 22	37/Female	HPA, LN , bone	Secondary hypothyroidism: FT4 0.774 ng/dL, TSH 0.176 μIU/mL; TPO-Ab (-), TG-Ab (-); Tg 1.6 ng/mL	N	6.1	Right thyroidectomy followed by MA*6	No signs of active disease after 7 years
Case 23	38/Male	HPA, lung, LN, skin	Secondary hypothyroidism: FT4 0.622 ng/dL, TSH 0.35 μIU/mL; TPO-Ab (-), TG-Ab (-); Tg 22.7 ng/mL	Nodular type	Normal	MA*6	Recurrence after 1 year 7 months
Case 24	40/Male	HPA, lung, LN, bone	Euthyroidism: FT4 0.921 ng/dL, TSH 3.973 μIU/mL; TPO-Ab (-), TG-Ab (-); Tg 296.3 ng/mL	Nodular type	Elevated (unknown value)	Right thyroidectomy followed by MA*5	Recurrence after 2 years 9 months
Case 25	40/Female	HPA, skin	Secondary hypothyroidism: FT4 0.701 ng/dL, TSH 0.372 μIU/mL; TPO-Ab (+), TG-Ab (+)	Diffuse type	N	MA*6	Lost follow-up
Case 26	47/Female	HPA, lung, liver	Secondary hypothyroidism: FT4 0.54 ng/dL, TSH 0.07 μIU/mL; TPO-Ab (-), TG-Ab (-)	Nodular type	N	MA*6	Recurrence after 6 months, treated with cladribine and TCD, alive after 3 years
Case 27	50/Female	HPA, bone, skin	Subclinical hypothyroidism: FT4 0.836 ng/dL, TSH 6.278 μIU/mL; TPO-Ab (-), TG-Ab (-); Tg 5.6 ng/mL	Nodular type	N	MA*6, ongoing TCD	No signs of active disease after 5 years

SUV, standardized uptake values; HPA, hypothalamic-pituitary axis; LN, lymph nodes; FT4, free thyroxine; TSH, thyroid stimulating hormone; Tg, thyroglobulin; TPO-Ab, anti-thyroid peroxidase antibody; TG-Ab, anti-thyroglobulin antibody; N, not available; MA, methotrexate & cytarabine; CVOP, cyclophosphamide & vindesine & prednisone & etoposide; CEOP, cyclophosphamide & vinblastine & prednisone & etoposide; GDP-ML, gemcitabine & dexamethasone & cisplatin & methotrexate; TCD, thalidomide & cyclophosphamide & dexamethasone.

Case 1: the reference range of FT4 is within 0.89-1.76 ng/dL, the reference range of TSH is within 0.64-6.27 μIU/mL.

Case 5: the reference range of FT4 is within 0.75-1.52 ng/dL, the reference range of TSH is within 0.4-5.6 μIU/mL.

Case 9: the reference range of FT4 is within 0.93-1.71 ng/dL, the reference range of TSH is within 0.27-4.2 μIU/mL.

Case 2, 4, 6, 8, 10-14, 16-27: the reference range of FT4 is within 0.81-1.89 ng/dL, the reference range of TSH is within 0.38-4.34 μIU/mL.

The reference range of thyroglobulin is within 1.4-78.0 ng/mL.

**Table 2:** Characteristics of LCH patients with and without thyroid involvement.

	Children with thyroid involvement (N = 7)	Children without thyroid involvement (N = 43)	p value	Adult with thyroid involvement (N = 20)	Adult without thyroid involvement (N = 178)	p value
Median age, years (range)	15 (8-16)	12.9 (1.3-17)	0.15	24 (19-50)	33 (19-79)	0.011
Sex, male, % (n)	71.4 (5)	60.5 (26)	0.579	50.0 (10)	64.0 (114)	0.218
Systemic involvement						
HPA -%(n)	100 (7)	62.8 (27)	0.05	95 (19)	42.1 (75)	<0.001
Lung -%(n)	85.7 (6)	25.6 (11)	0.004	70 (14)	50.6 (90)	0.099
Lymph node -%(n)	57.1 (4)	14.0 (6)	0.023	50 (10)	23.6 (42)	0.011
Bone -%(n)	14.3 (1)	55.8 (24)	0.049	45 (9)	73.6 (131)	0.008
Liver -%(n)	14.3 (1)	14.0 (6)	1	30 (6)	14.6 (26)	0.103
Thyroid function status	N = 5	N = 29	0.002	N = 19	N = 114	<0.001
Euthyroidism-%(n)	20.0 (1)	69.0 (20)	<0.05	31.6 (6)	83.3 (95)	<0.05
Primary hypothyroidism-%(n)	60.0 (3)	0 (0)	<0.05	21.1 (4)	2.6 (3)	<0.05
Secondary hypothyroidism-%(n)	0 (0)	20.7 (6)	0.559	31.6 (6)	9.6 (11)	<0.05
Subclinical hypothyroidism-%(n)	20.0 (1)	10.3 (3)	0.128	15.8 (3)	4.4 (5)	<0.05
Thyroid ultrasound	N = 5	N = 22		N = 15	N = 34	
Normal thyroid ultrasonography-%(n)	0 (0)	54.5 (12)	0.047	(0)	55.9 (19)	<0.001
F-18-FDG PET/CT	N = 3	N = 12		N = 12	N = 84	
Elevated FDG accumulation in the thyroid region-%(n)	100 (3)	0 (0)	0.002	91.7 (11)	4.8 (4)	<0.001

F-18-FDG PET/CT, 18-F-fluorodeoxyglucose positron emission tomography/computed tomography; HPA, hypothalamic-pituitary axis.

### Clinical manifestations

The most common initial complaint in patients with thyroid LCH was diabetes insipidus (DI), observed in four pediatric (57.1%) and 18 (90.0%) adult patients. In addition, two pediatric patients presented with short stature (related to growth hormone deficiency) and secondary amenorrhea (related to hypogonadotropic hypogonadism) as the initial manifestation, respectively.

Many patients developed clinical manifestations related to thyroid involvement. Fourteen (51.9%) patients presented with neck swelling or mass; of these, two patients progressed to dyspnea, and one was accompanied by neck pain. In addition, symptoms of hypothyroidism, such as fatigue and memory loss, were observed in three cases.

All 27 patients with thyroid LCH had a multi-system disease. Except for the thyroid, the most affected system was the HPA, followed by the lungs and lymph nodes. The involvement of risk organs was observed in eight cases, seven of which had liver involvement. Patients with thyroid LCH had higher frequencies of HPA (children: 100% vs. 62.8%, P=0.05; adult: 95% vs. 42.1%, P<0.001), lung (children: 85.7% vs. 25.6%, P=0.004; adult: 70% vs. 50.6%, P=0.099), and lymph node involvement (children: 57.1% vs. 14%, P=0.023; adult: 50% vs. 23.6%, P=0.011) and a lower frequency of bone involvement (children: 14.3% vs. 55.8%, P=0.049; adult: 45% vs. 73.6%, P=0.008) than those without thyroid gland involvement (**Table 2**).

### Laboratory findings

Twenty-four (5 children and 19 adults) patients with thyroid LCH underwent thyroid profile evaluation at diagnosis. Seven (29.2%) had normal thyroid function, 7 (29.2%) had primary hypothyroidism, 4 (16.7%) had subclinical hypothyroidism, and 6 (25.0%) had secondary hypothyroidism. The thyroid function status of children and adults are described separately in **Table 1**. Three patients with normal thyroid function were reexamined regularly and maintained a normal thyroid status. In addition, seven patients with primary hypothyroidism and two with subclinical hypothyroidism were regularly followed up; three gradually progressed to secondary hypothyroidism, and the rest remained largely unchanged. All the patients with hypothyroid status required long-term levothyroxine therapy.

The thyroid function of LCH patients with and without thyroid involvement were compared separately for children and adults (**Table 2**). Thyroid LCH patients had a higher frequency of primary hypothyroidism (children: 60% vs. 0%, P<0.05; adult: 21.1% vs. 2.6%, P<0.05) and a lower frequency of euthyroidism (children: 20% vs. 69%, P<0.05; adult: 31.6% vs. 83.3%, P<0.05) than patients without thyroid involvement (**Table 2**).

Twenty-one patients were assessed for anti-thyroid peroxidase (TPO-Ab) and anti-thyroglobulin (TG-Ab) antibodies. Three tested positive for both, whereas two tested positive for TG-Ab only. Eleven patients underwent screening for thyroglobulin (in peripheral blood or thyroid aspiration fluid). Of these, 5 (45.5%) presented elevated thyroglobulin levels.

### Thyroid ultrasonography

**Figure 1:**
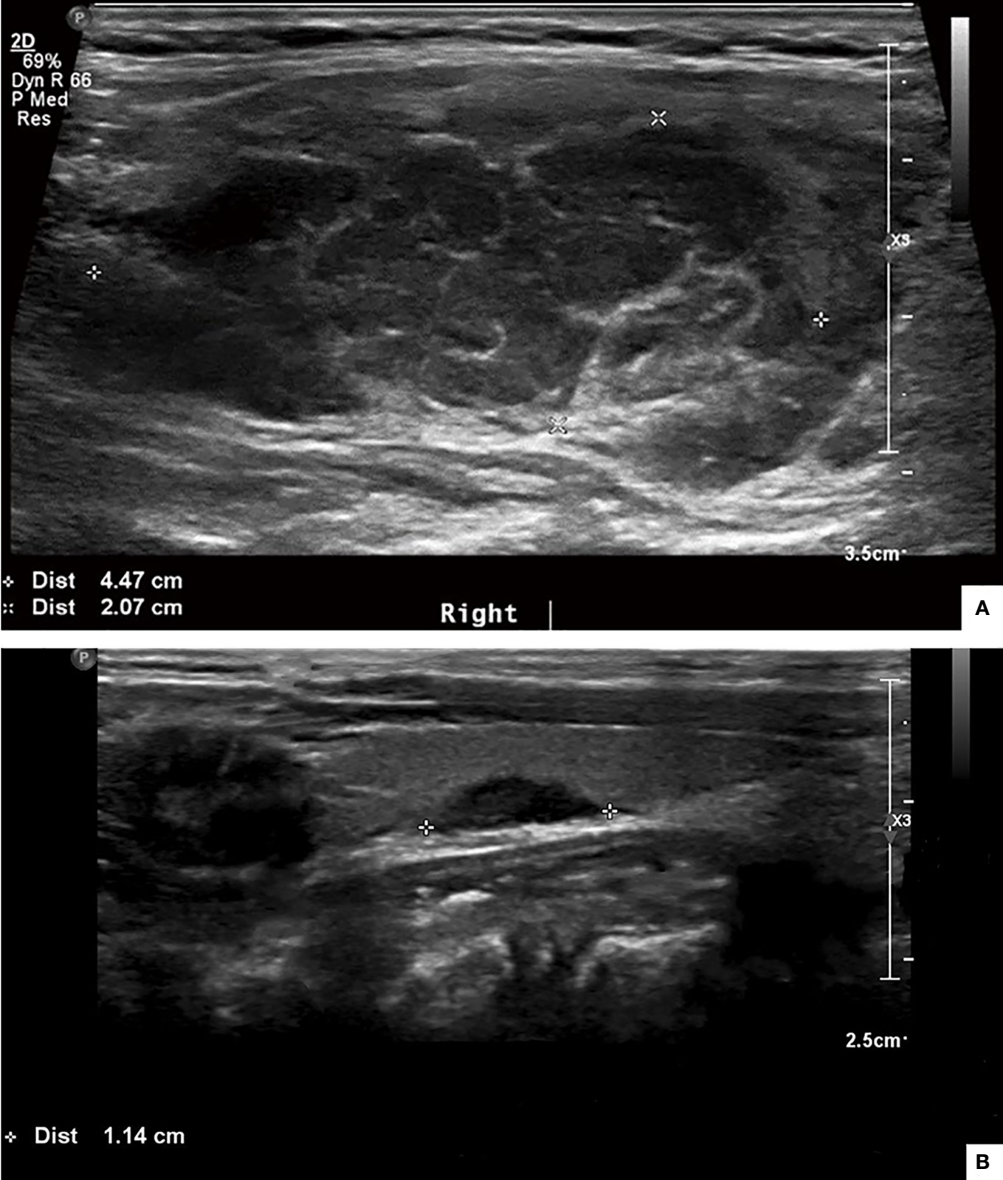
Two typical ultrasonographic types of thyroid LCH. The diffuse type: a large hypoechoic area covering 80%-90% of the right thyroid lobe **(A)**. The nodular type: a hypoechoic nodule with irregular contour on the left thyroid lobe **(B)**.

Twenty patients underwent thyroid ultrasound, and all had abnormal findings. All ultrasonographic features are summarized in **Table 3**. Two major ultrasonographic types were observed: diffuse (n=11) and nodular type (n=9) (**Figure 1**). The diffuse type refers to more diffuse lesions, presenting as the diffuse enlargement of the thyroid gland, a large hypoechoic area covering the unilateral lobe or the entire gland, or a large amount of patchy hypoechoic areas partly fused. The nodular type refers to the lesions manifested as single or multiple hypoechoic nodules. In addition, the hypoechoic areas or nodules were often irregular in shape, with regular or irregular borders and rare calcifications, and were frequently accompanied by cervical lymphadenopathy. The hypoechoic areas or nodules were generally large, with the longest area diameters of 1.1 cm–5.8 cm.

**Table 3:** Ultrasonographic features of thyroid involvement of LCH.

Ultrasound imaging	Casenumber	Size and location	Echogenicity	Calcification	Contour	Border	Blood flow signals	Enlarged lymphnodes in the neck
Diffuse type (n=11)	Case 5	cover the entire gland	Hypo	–	–	–	abundant	Y
	Case 12	cover 80%-90% of the right lobe and 50% of the left lobe	Hypo	–	NA	NA	normal	Y
	Case 16	0.4×0.3 cm on the right; cover almost the entire left lobe	Hypo	–	NA	NA	normal	Y
	Case 17	cover bilateral middle and lower lobes and isthmus	Hypo	–	–	–	abundant	N
	Case 25	4.5×2.1 cm on the right; cover almost the entire left lobe	Hypo	–	NA	NA	NA	Y
	Case 20	5.8×1.9×1.5 cm on the right; 4.6×1.9×1.3 cm on the left	Hypo	–	NA	NA	NA	Y
	Case 1	diffused enlargement of the gland	–	–	–	–	–	–
	Case 13	diffused enlargement of the gland	–	–	–	–	–	–
	Case 3	multiple patchy hypoechoic areas	Hypo	–	–	–	NA	NA
	Case 14	multiple patchy hypoechoic areas	Hypo	–	–	–	NA	NA
	Case 21	multiple patchy hypoechoic areas	Hypo	–	–	–	NA	NA
Nodular Type (n=9)	Case 2	1.3×0.5×0.4 cm on the left	Hypo	N	irregular	not clear	normal	N
	Case 4	2.9×1.7×3.8 cm on the right; solid nodules in left lobes	Hypo	NA	NA	NA	NA	NA
	Case 23	1.1×0.6 cm on the right; 2.7×1.0 cm on the left	Hypo	–	irregular	NA	normal	N
	Case 24	3.2×3.6×2.3 cm on the right; 0.6×0.4 cm on the left	Medium to hyper	Y (combined PTC)	irregular	not clear	abundant	Y
	Case 26	0.8×0.8×0.6 cm on the right; 1.2×0.6×0.5 cm on the left	Hypo	N	irregular	clear	normal	N
	Case 27	0.7×0.4 cm on the right; 0.5×0.3 cm on the left	Hypo	N	regular	clear	normal	N
	Case 9	Solid nodules in both lobes	NA	NA	NA	NA	NA	NA
	Case 11	solid nodules in left lobe	NA	NA	NA	NA	NA	NA
	Case 19	multiple nodules in left lobe	Hypo	NA	NA	NA	NA	NA

HPA, hypothalamic-pituitary axis. NA, not available; Y, yes; N, none; PTC, papillary thyroid carcinoma.

In LCH patients without thyroid involvement, 22 children and 34 adults underwent thyroid ultrasound; 54.5% of the children and 55.9% of the adults had normal thyroid ultrasonography (**Table 2**). Among the 10 children and 15 adult patients with abnormal thyroid ultrasonography, 8 children and 11 adults had multi-cysts in the thyroid gland. In comparison, 2 children and 4 adults had multiple hypoechoic nodules with the longest diameter of 0.3 cm–1.4 cm.

### 18-F-fluorodeoxyglucose positron emission tomography/computed tomography

Fifteen patients underwent 18-F-FDG PET/CT before the primary disease treatment. Fourteen displayed increased FDG accumulation in the thyroid region, with standardized uptake values (SUV max) of 5.3–12.8 (**Table 1**). Declined or lack of FDG accumulation was observed in 8 patients who repeated 18-F-FDG PET/CT after receiving systemic therapy.

In LCH patients without thyroid involvement, 12 children and 84 adults underwent 18-F-FDG PET/CT, and few patients displayed elevated SUV in the thyroid region. The positive rate of hypermetabolic thyroid regions on PET/CT was higher in patients with thyroid involvement than in those without thyroid involvement (children: 100% vs. 0%, P=0.002; adults: 91.7% vs. 4.8%, P<0.001).

### Pathological findings

**Figure 2:**
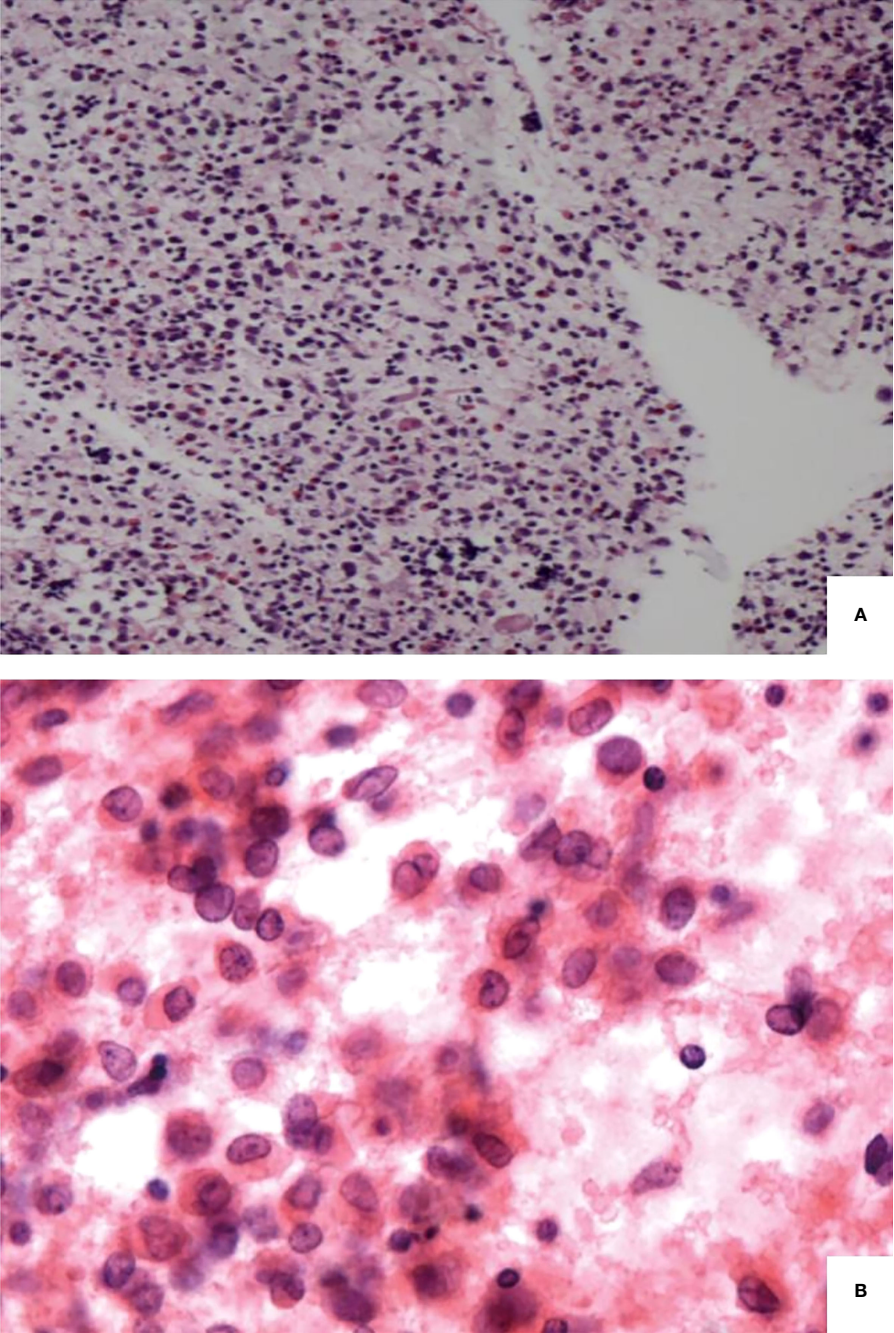
Pathological findings of langerhans cell histiocytosis. Langerhans cells admixed with eosinophils and chronic inflammatory cells **(A)**. Langerhans cells with a low nuclear-to-cytoplasmic ratio, irregular nuclear contour and prominent nuclear grooves **(B)**.

Almost all thyroid specimens displayed histopathological features, with dense polymorphic cellular infiltrates that comprised Langerhans histiocytes, numerous eosinophils, and chronic inflammatory cells (**Figure 2A**). The Langerhans histiocytes were scattered and loosely cohesive medium-sized cells with a low nuclear-to-cytoplasmic ratio, abundant finely vacuolated cytoplasm, irregular nuclear contours, and prominent nuclear grooves (**Figure 2B**). The immunohistochemical results of 20 patients were available. Additionally, the positive expression rates of S100, CD1a, langerin, and CD68 were 100% (22/22), 100% (22/22), 85.7% (6/7), and 93.3% (14/15), respectively.

### Diagnostic modality

**Figure 3:**
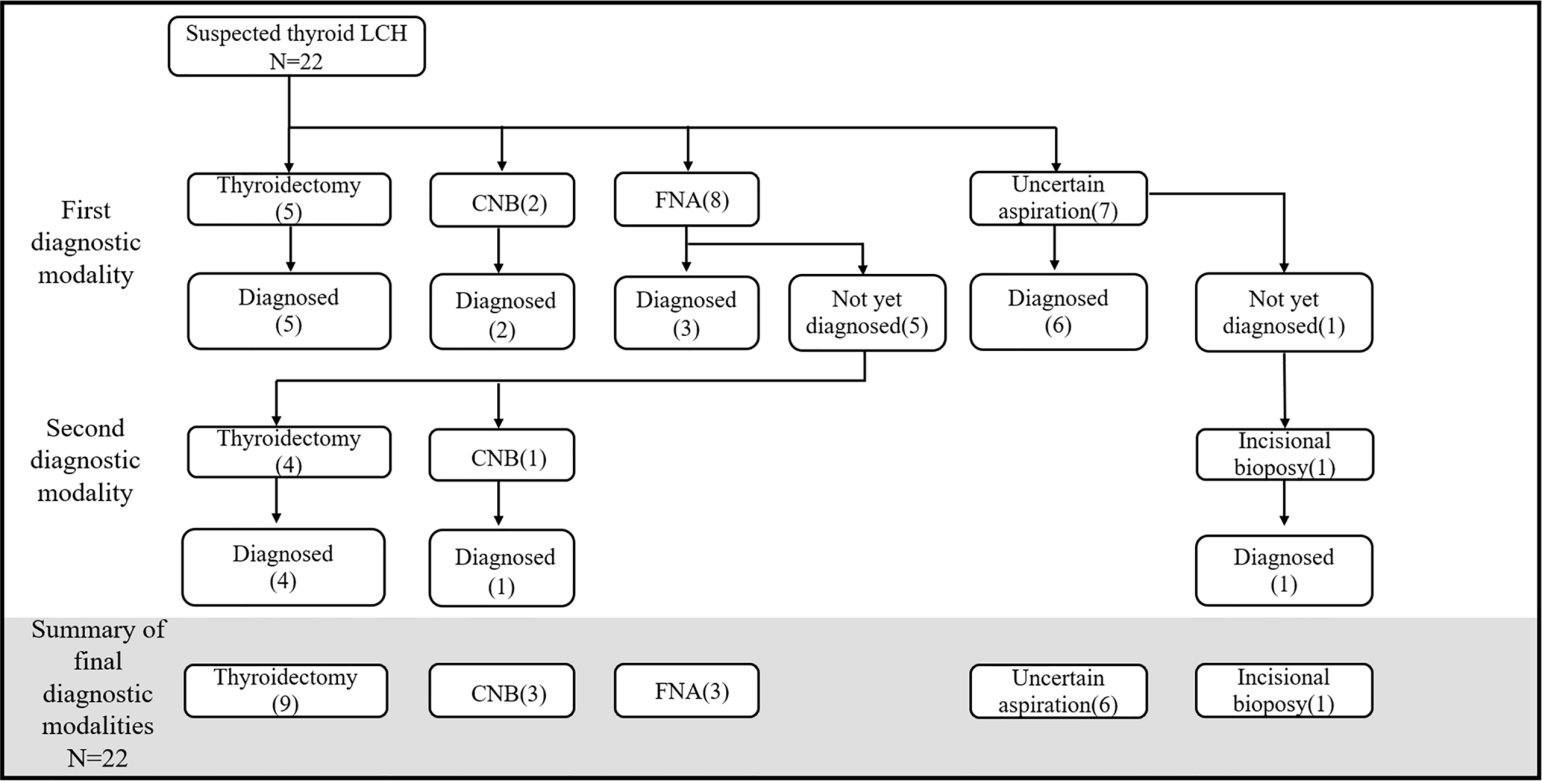
Diagnostic process of thyroid LCH. CNB, core-needle biopsy; FNA, fine-needle aspiration; Uncertain aspiration, the gauge of the needle was not described.

The methods used to obtain thyroid specimens included surgery and ultrasound-guided fine needle aspiration (FNA) or core needle biopsy (CNB). Seventeen (77.3%) patients underwent thyroid aspiration as their first diagnostic examination, and the remaining 5 (22.7%) underwent thyroidectomy. The diagnosis was confirmed in 54.5% of patients by thyroid aspiration and in 45.5% by thyroid surgery. The overall sensitivity of CNB or thyroid surgery was 100%, whereas that of FNA was 37.5% (**Figure 3**).

The reasons for choosing thyroidectomy as the first diagnostic modality were identified in three cases: dyspnea due to goiter (n=2) and a combination of primary hyperparathyroidism (n=1).

Five patients could not be diagnosed with LCH after thyroid FNA. The reasons were (1): insufficient materials for immunohistochemistry (n=2), (2) complicated conditions with concomitant papillary thyroid carcinomas (PTC) (n=2), and (3) misdiagnosis of chronic inflammation (n=1). However, the diagnosis of LCH was subsequently confirmed after thyroid surgery (n=4) and CNB (n=1).

### Treatment and prognosis

The treatments and prognoses of the patients are summarized in **Table 1**. All 27 patients received systemic chemotherapy, and the main drugs were methotrexate and cytarabine. Of the 27 patients, 12 (44.4%) reported no signs of active disease after treatment, with the disease-free follow-up duration ranging from 1 year 5 months to 9 years, 5 (18.5%) were lost to follow-up, and 9 (33.3%) experienced disease recurrence or progression after the initial treatment. Five of the six patients with risk organ involvement who were followed up experienced disease recurrence or progression.

### Thyroid Langerhans cell histiocytosis together with papillary thyroid carcinoma

The characteristics of both cases of thyroid LCH with PTC (cases 8 and 24) are summarized in **Table 1**.

## Discussion

Thyroid involvement in LCH was previously believed to be rare. With the exception of a case series of 7 patients reported by Thompson et al. (7) and a case series of 36 adult patients reported by Cai (8), LCH has only been reported as individual cases to date. Here, we reported a 10.89% incidence of thyroid involvement in LCH, suggesting that thyroid LCH is not as rare as previously presumed.

Epidemiological data have demonstrated that LCH is more common in children than adults (3, 9, 10). However, previous case reports of LCH with thyroid involvement have been predominantly in adults and less commonly in children (**Table 4**). Our results revealed that the incidence of thyroid LCH in children was slightly higher than in adults, suggesting that more attention should be paid to thyroid involvement in children with LCH.

**Table 4:** Demographic characteristics of patients reported in literatures.

	Cases, n	Adlut : child ratio	The range of age	Mean age, years	Male : female ratio	Author
Single center cohort	7	6 : 1	2 months to 55 years	33.7	1 : 1.33	Thompson et al. 1996 (7)
Literature review	66	2.7 : 1	5 months to 61 years	NR (median age 28)	1 : 1.44	Patten et al. 2012 (11)
Literature review	29	6.3 : 1	3 years to 73 years	NR	1 : 1.27	Zhang et al. 2021 (12)

NR, not reported.

In previous literature, thyroid involvement mostly occurred as part of multi-system LCH (8, 12, 13), consistent with our results. LCH patients with thyroid involvement present a series of clinical manifestations that are different from those of general LCH: (1) approximately half of the patients develop neck-related symptoms; (2) most patients show DI as the first clinical sign; (3) the frequency of systemic involvement varies, with HPA involvement being the most common rather than bone involvement.

Thyroid involvement in LCH usually manifests itself as neck swelling or mass. Patten et al. reported that patients with thyroid LCH present with diffuse (59%) or nodular thyroid (25.8%) enlargement (11). Xia et al. reported goiters or neck masses (76.3%) as the common clinical features (14). Furthermore, neck pain and obstructive symptoms like dyspnea are rare. Zhang reported a rare case of painful neck swelling and progressive dyspnea diagnosed as a spontaneous thyroid hemorrhage (12).

As previously reported, it is common to identify posterior pituitary dysfunction in LCH. Sagna et al. observed that DI occurred in 55.3% of patients with LCH (15). Few reports have noticed a much higher frequency of DI in patients with thyroid LCH (16). In our cohort, the proportion of patients with DI was high. In addition, many patients presented other manifestations of HPA involvement, such as secondary adrenal insufficiency and hypogonadotropic hypogonadism. Our results reveal that LCH thyroid involvement is more prone to coexist with HPA involvement, especially DI; however, the underlying mechanism is unknown. Cai observed that more LCH patients with thyroid involvement have BRAFN486_P490 mutations than those without thyroid involvement (8), which may partially explain the higher frequency of pituitary involvement in thyroid LCH. It is worth exploring the specific reasons in future studies. Besides, thyroid LCH differs from general LCH in the involvement of other systems. For instance, bone involvement is less common in thyroid LCH, whereas the lung is more commonly involved. These findings indicate that systemic evaluations, particularly of HPA and pulmonary involvement, should be performed once thyroid LCH is diagnosed.

As mentioned above, thyroid involvement in LCH is not as rare and is usually accompanied by DI. Regarding the diagnostic methods, thyroid aspiration is easier and safer than pituitary stalk and lung biopsy. Therefore, increasing the awareness of thyroid involvement in LCH is vital for accurate diagnosis. In our study, stalk biopsy was considered in a few cases to obtain pathological evidence, which was needed for subsequent therapy. In five cases, pathological diagnostic evidence was not obtained from thyroid lesions but those in the lung, bone, cervical lymph nodes, and skin. This indicates that if the possibility of thyroid involvement is identified, a subset of patients could be exempted from riskier and more invasive surgical procedures, thereby reducing the difficulty of LCH diagnosis.

As for thyroid function status, it has been reported that 37.9–40.9% of patients have euthyroidism, 19.7%–31.03% have hypothyroidism, 6.9%–10.6% have subclinical hypothyroidism, and 1.5%–3.45% have hyperthyroidism (11, 12). Our results largely parallel these reports. Furthermore, we observed that thyroid LCH patients had a higher frequency of primary hypothyroidism and a lower frequency of euthyroidism than patients without thyroid involvement. This finding suggests that primary hypothyroidism may imply thyroid involvement in patients suspected of multi-system LCH. Interestingly, we observed that the probability of maintaining a normal thyroid function is high if thyroid function is normal before treatment. In addition, the positive rate of TPO-Ab and TG-Ab in thyroid LCH was low, the significance of which was not determined. Few reports focus on the level of thyroglobulin, whereas approximately half of the patients in our study whose thyroglobulin were evaluated presented elevated results. Therefore, we suggest paying more attention to thyroglobulin levels before and after therapy.

Ultrasound is the most common noninvasive modality used to evaluate thyroid diseases. In our cohort, all the patients with thyroid LCH had abnormal findings on thyroid ultrasound. In contrast, approximately half of the LCH patients without thyroid involvement presented normal thyroid ultrasonography, and most of the remaining half manifested as multi-cysts on the thyroid gland. This suggests that most patients with thyroid LCH have abnormal ultrasound findings. And learning the ultrasonographic features of thyroid LCH can help to differentiate it from other thyroid disorders. Chen et al. summarized the ultrasonographic features of 12 reported cases of thyroid LCH. They observed that the main features in the ultrasonograms were isolated or multiple hypoechoic nodules with rare calcifications (17). Our study observed two major ultrasonographic types of thyroid LCH. The diffuse type manifests as diffuse enlargement of the thyroid gland, a large hypoechoic area covering the gland, or a large amount of patchy hypoechoic areas partly fused with each other. The second is the nodular type, which manifests as single or multiple hypoechoic nodules. For patients suspected of multi-system LCH, thyroid involvement should be considered if the thyroid ultrasonography displays these features.

However, thyroid involvement in LCH can still be misdiagnosed as Hashimoto thyroiditis or thyroid cancer in some cases. Therefore, detailed descriptions of the contours and borders of the hypoechoic areas/nodules’ characteristic of thyroid LCH are needed (18). We analyzed this issue and concluded that most hypoechoic areas/nodules are irregular in shape, with well-defined or poorly defined borders. These findings differed from the features of thyroid tumors, such as papillary carcinoma. In previous studies and our research (17), there were barely any calcifications in the hypoechoic nodules observed in thyroid LCH. Interestingly, a patient in our cohort with calcified thyroid nodules was pathologically confirmed to have both papillary thyroid carcinoma and thyroid LCH. Therefore, LCH patients with calcification on thyroid ultrasound should be alert to the possibility of thyroid cancer.

18-F-FDG PET/CT is considerably useful for evaluating systemic involvement in LCH (19). Many studies have indicated that the involved thyroid gland presents elevated FDG accumulation, with the reported SUV max values of 5.3–14.7 (19–25). These findings are consistent with those of this study. Moreover, our study observed that most LCH patients without thyroid involvement presented no FDG accumulation in the thyroid region. Therefore, we recommend considering PET/CT evaluation for patients with suspected thyroid LCH, particularly when inconclusive cytological results are obtained. In addition, a thyroid gland SUVmax ≥ 5 is appropriate for diagnosing LCH.

Several cases reported the role of FNA cytology (FNAC) in diagnosing thyroid LCH (26, 27). For patients suspected of LCH, FNAC is a much easier and safer diagnostic procedure than hypophysis biopsy. However, the diagnosis is sometimes ambiguous or incorrect due to the limited amount of biopsy tissue (28, 29). In our study, the sensitivity of FNA was only 37.5%. However, this value may be underestimated because the needle gauges used in seven cases were unknown. Therefore, we believe that FNA is useful for diagnosing thyroid LCH but is also limited in some situations. In this regard, core needles and surgical biopsies are of vital importance. Moreover, it should be noted that a thyroid surgical biopsy could also yield a wrong diagnosis. In several cases, the diagnosis of PTC confirmed through surgical biopsy was corrected to LCH due to the identification of disseminated disease (30, 31). Overall, we recommend FNA for patients with suspected thyroid LCH. If the diagnosis is not established after investigating cytomorphological features and immunocytochemistry results, CNB or surgical incision should be considered as a more sensitive diagnostic modality. Meanwhile, evidence of a systemic disease also helps in diagnosing LCH.

No specific guidelines are available for managing thyroid LCH. In general, the treatment options vary depending on whether the case is that of solitary thyroid involvement or a multi-system disease. Most patients with isolated thyroid LCH are treated with surgical resection alone (32). However, the optimal choice of surgical technique is currently inconclusive, and subtotal, near total, or total thyroidectomy can be selected as an option (33). Postoperatively, many patients do not receive further treatment and experience no recurrence or progression during follow-up (7, 34). Attakkil et al. reported an eight-year-old male with LCH confined to the thyroid accepted chemotherapy and had a satisfactory prognosis. They emphasized the role of chemotherapy in preserving thyroid function (35). For patients diagnosed with multi-system LCH, chemotherapy should be the first choice. Cytarabine-, cladribine-, and vinblastine-based regimens are preferred as common treatment options (1, 2). The efficacy of short-term chemotherapy using MTX, doxorubicin, cyclophosphamide, vincristine, prednisone, and bleomycin in aggressive adult LCH has been demonstrated (36). Wang designed thalidomide combined with dexamethasone and cyclophosphamide as an oral administration regimen for recurrent or refractory adult LCH patients (37). Notably, chemotherapy combined with surgery is also an option, particularly for those with multi-system disease and obstructive symptoms. In recent years, more studies have reported the efficacy of targeted therapies, such as BRAF and MAPK pathway inhibitors (1).

Thyroid LCH combined with PTC is not rare, with 2 cases in our cohort and nearly 15 cases reported in literature (32, 38–46), suggesting raising awareness for coexisting PTC and thyroid LCH.

BRAF is a serine/threonine kinase involved in cell survival, proliferation, and differentiation (47). The BRAF gene mutations have a particularly high frequency in thyroid cancer and LCH. The average frequency of BRAF mutations is 44% in PTC (48). Furthermore, the mutation of oncogenic BRAF V600E (the majority of BRAF mutations) occurs in approximately 50% of LCH individuals (49). Many cases of thyroid LCH complicated by PTC paid attention to the BRAF mutation. Ozisik et al. reported BRAF V600E in PTC and LCH tissues (46). However, Hamad et al. observed BRAF V600E and BRAF V600K mutations in PTC and LCH tissues, respectively (45). These findings indicate a possible etiological relationship between both disorders and remind us to investigate PTC in LCH patients with BRAF mutations.

There is currently no standard treatment for thyroid LCH with PTC. In previous studies, most patients underwent surgery, and those with multi-system LCH lesions received systemic therapy. However, Vergez et al. advocate surgical removal because they treated a patient with a huge neck mass who did not undergo surgery and died of acute pulmonary distress due to a considerable increase in the size of the mass after three chemotherapies (41).

## Conclusion

Thyroid involvement in LCH has not been fully studied. Therefore, our study provided a comprehensive evaluation of thyroid LCH in children and adults and observed that thyroid LCH is not rare, contrary to previous notions. Identifying thyroid involvement is essential for LCH diagnosis because information on thyroid histopathology is readily available. The possibility of thyroid LCH should be carefully investigated if a patient presents symptoms of DI or neck swelling or mass, primary hypothyroidism, diffuse lesions or hypoechoic nodules on thyroid ultrasound, and evidence of systemic involvement. Thyroid aspiration and 18-F-FDG PET/CT can effectively assist in diagnosis. For patients diagnosed with thyroid LCH, attention should be paid to the involvement of the HPA and lungs.

## Data availability statement

The original contributions presented in the study are included in the article/Supplementary Material. Further inquiries can be directed to the corresponding authors.

## Ethics statement

The studies involving human participants were reviewed and approved by Peking Union Medical College Hospital Ethics Committee (No. I-22PJ108). Written informed consent to participate in this study was provided by the participants’ legal guardian/next of kin.

## Author contributions

YL: data collection, data analysis, interpretation of the results, and writing. XC, HL, and HY: data collection and interpretation of the results. LC, YX, LH, and HZ: data curation and interpretation of the data. NL and XL: conception and design of the study, review, and editing. All authors contributed to the article and approved the submitted version.

## Funding

This work was supported by the Chinese Academy of Medical Sciences (CAMS) Innovation Fund for Medical Science (CIFMS; No. 2021-1-I2M-022).

## Acknowledgments

We thank all patients in this study. We could not have completed this study without their participation.

## Conflict of interest

The authors declare that the research was conducted in the absence of any commercial or financial relationships that could be construed as a potential conflict of interest.

## Publisher’s note

All claims expressed in this article are solely those of the authors and do not necessarily represent those of their affiliated organizations, or those of the publisher, the editors and the reviewers. Any product that may be evaluated in this article, or claim that may be made by its manufacturer, is not guaranteed or endorsed by the publisher.
